# Implantable Osmotic Transport Device Can Reduce Edema After Severe Contusion Spinal Cord Injury

**DOI:** 10.3389/fbioe.2020.00806

**Published:** 2020-07-10

**Authors:** Christopher Hale, Jennifer Yonan, Ramsey Batarseh, Roman Chaar, Carrie R. Jonak, Shaokui Ge, Devin Binder, Victor G. J. Rodgers

**Affiliations:** ^1^Department of Bioengineering, University of California, Riverside, Riverside, CA, United States; ^2^Division of Biomedical Sciences, School of Medicine, University of California, Riverside, Riverside, CA, United States; ^3^Division of Biostatistics & Bioinformatics, School of Medicine, University of California, Riverside, Riverside, CA, United States

**Keywords:** spinal cord injury, edema, severe contusion, implantable device, membrane separations, osmotic transport device (OTD)

## Abstract

Recent findings from the ISCoPe study indicate that, after severe contusion to the spinal cord, edema originating in the spinal cord accumulates and compresses the tissue against the surrounding dura mater, despite decompressive laminectomy. It is hypothesized that this compression results in restricted flow of cerebrospinal fluid (CSF) in the subarachnoid space and central canal and ultimately collapses local vasculature, exacerbating ischemia and secondary injury. Here we developed a surgically mounted osmotic transport device (OTD) that rests on the dura and can osmotically remove excess fluid at the injury site. Tests were performed in 4-h studies immediately following severe (250 kD) contusion at T8 in rats using the OTD. A 3-h treatment with the OTD after 1-h post injury significantly reduced spinal cord edema compared to injured controls. A first approximation mathematical interpretation implies that this modest reduction in edema may be significant enough to relieve compression of local vasculature and restore flow of CSF in the region. In addition, we determined the progression of edema up to 28 days after insult in the rat for the same injury model. Results showed peak edema at 72 h. These preliminary results suggest that incorporating the OTD to operate continuously at the site of injury throughout the critical period of edema progression, the device may significantly improve recovery following contusion spinal cord injury.

## Introduction

It is estimated that between 1/4 to 1/2 million people will endure a spinal cord injury (SCI) each year, world-wide (World Health Organization and International Spinal Cord Society, 2013). SCI causes long-lasting and often irreversible changes in motor, sensory and autonomic function, leading to reduced quality of life and increased morbidity rates in those affected (Barker et al., 2009; Hagen et al., 2010). SCI is characterized by the initial injury due to trauma, and by secondary cellular events that result in a further tissue damage. The period of secondary injury is accompanied by breakdown of the blood-spinal cord barrier (BSCB), hemorrhage, edema, ischemia, inflammation, and tissue necrosis at and around the injury site (Whetstone et al., 2003; Norenberg et al., 2004; Borgens and Liu-Snyder, 2012).

Edema levels (cytotoxic, vasogenic, or both) increase within the first few hours after injury (Leypold et al., 2008) and are correlated with poorer neurological outcome and reduced independence (Flanders et al., 1990, 1996, 1999). Larger increases in edema levels are observed in individuals with more severe injuries and reduced recovery following injury (Shepard and Bracken, 1999; Boldin et al., 2006; Bozzo et al., 2011). Spinal cord edema is also associated with both cord swelling and compression (Miyanji et al., 2007) which has been correlated with worse neurological outcome (Werndle et al., 2014; Papadopoulos, 2015; Phang and Papadopoulos, 2015). Unfortunately, surgical decompression and stabilization do not reduce edema or minimize the resulting ischemia-induced necrosis (Saadoun and Papadopoulos, 2010). In addition, its use in various SCI models along with its window of effectiveness remain controversial (Fehlings and Perrin, 2006). Further, the use of methylprednisolone (MP) to reduce edema and ischemia is waning due to controversy over its beneficial and harmful effects (Braughler and Hall, 1982; Hall et al., 1984; Cayli et al., 2004; Rozet, 2008). Still other research has looked into the beneficial effects of hypertonic saline (Nout et al., 2009) and the use of a mechanical tissue resuscitation device (Zheng et al., 2015) to minimize histological damage.

Recently, a series of significant clinical data in the Injured Spinal Cord Pressure Evaluation (ISCoPE) study has emerged indicating the importance of intraspinal pressure (ISP) at the injury site in outcome after SCI (Werndle et al., 2014; Papadopoulos, 2015; Phang and Papadopoulos, 2015; Phang et al., 2015; Varsos et al., 2015). These studies showed that: (i) ISP after SCI is elevated as the swollen cord is compressed against the dura; (ii) spinal cord perfusion pressure (SCPP) decreases at the site of injury and impacts outcome; and (iii) laminectomy with expansion duraplasty compared to decompressive laminectomy alone reduces ISP, increases SCPP, and leads to greater decompression of the injured cord (Phang et al., 2016; Chen et al., 2017; Chen et al., 2018; Gallagher et al., 2019; Hogg et al., 2019). These findings have also been corroborated in rodent and porcine models of SCI (Saadoun et al., 2008; Leonard et al., 2015; Khaing et al., 2017; Streijger et al., 2017). These initial studies suggest that spinal cord parenchymal swelling due to edema accumulation continues to expand radially until the tissue reaches the dura and can no long swell outward, despite routine decompressive laminectomy. This leads to an inevitable localized pressure build-up that causes the subarachnoid space to collapse at the epicenter and significant constriction of flow within local blood vessels (Soubeyrand et al., 2014a; Khaing et al., 2018; Saadoun and Papadopoulos, 2020). The collapsed blood vessels are no longer able to supply nutrients to the surrounding tissue and this creates local ischemia, further worsening tissue secondary injury (Gallagher et al., 2019).

These key new clinical data and recent animal models indicate the importance of developing innovative treatments aimed at preventing or reversing spinal cord edema and subsequent swelling following injury. To date there is no widely accepted and effective treatment for edema following SCI. It is widely accepted, however, that early intervention may limit the amount of secondary damage. There is, therefore, a need for new methods to effectively ameliorate edema following SCI in order to minimize spinal cord compression, decrease ISP at the injury site, improve vascular perfusion (SCPP), and improve neurological outcome. In this work we develop our currently effective osmotic transport device (OTD) that has been shown to improve outcome in global and focal models of cerebral edema (McBride et al., 2012, 2014, 2016) and apply it to SCI in a well-accepted rodent model of thoracic contusion SCI.

We have recently demonstrated that through establishing an external osmotic gradient, water can be removed from the brain in a controlled manner under normal and pathological brain swelling conditions. We found that the OTD reduced tissue water content and dramatically improved neurological outcome in an acute mouse models of cytotoxic edema and traumatic brain injury (TBI induced by controlled cortical impact, CCI) without causing histological damage (McBride et al., 2012, 2014, 2016). These results established proof-of-principle for the concept of direct osmotherapy for treatment of CNS edema.

**FIGURE 1:**
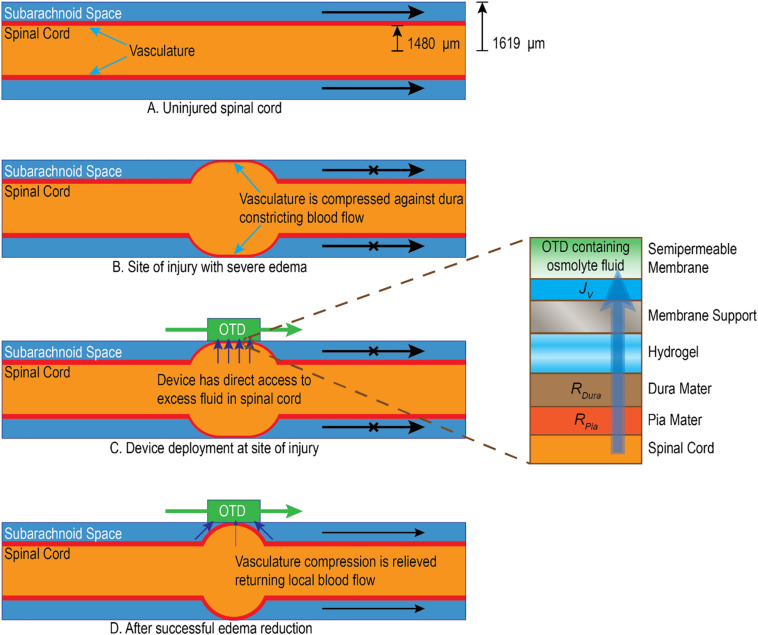
Simplified illustration of how edema impacts local spinal cord environment and the potential mechanism of action for the OTD. **(A)** Uninjured tissue has unobstructed vasculature and cerebrospinal fluid (CSF) in the subarachnoid space. **(B)** After injury, severe edema emanates from the central location of the spinal cord and forces tissue against the dura mater. This constricts local blood vessels in the subarachnoid space (illustrated as red) as well as reduces CSF movement in the local region (Saadoun and Papadopoulos, 2020). **(C)** The OTD is placed at the point of injury directly on the dura mater. Hydrogel is used to maintain a continuous aqueous interface. The OTD uses osmotic pressure to gently remove water across the permeable tissue (through the cord tissue, pia mater, and dura mater). **(D)** In time, the OTD may reduce the water content of the swollen tissue to alleviate pressure and constriction of the vasculature and allow fluid movement in the subarachnoid space. Estimates of typical tissue radii with cylindrical approximations for adult rat spinal cord are shown in panel **(A)** and are derived from the literature (Soubeyrand et al., 2014a).

We hypothesize that a similar OTD, placed on the dura mater of the spinal cord at the site of injury, can withdraw fluid from the cord by permeation through the adjacent tissue. The expectation is that the reduced swelling could provide relief of vasculature compression. Figure 1 provides a simplified model of the dynamics of tissue compartments in SCI and how the OTD is proposed to ameliorate SCI.

In 4-h blunt trauma SCI studies with rats (OTD applied one hour after injury followed by 3 h of operation), we showed that our spinal cord OTD significantly reduces edema as determined by tissue water content at the injury site. We further estimated the importance of this reduction and hypothesize that this swelling reduction may significantly open flow in the subarachnoid space and spinal cord tissue itself, potentially reducing constrictions of the local vasculature.

## Results

### Progression of Edema After Severe Contusion at T8

**FIGURE 2:**
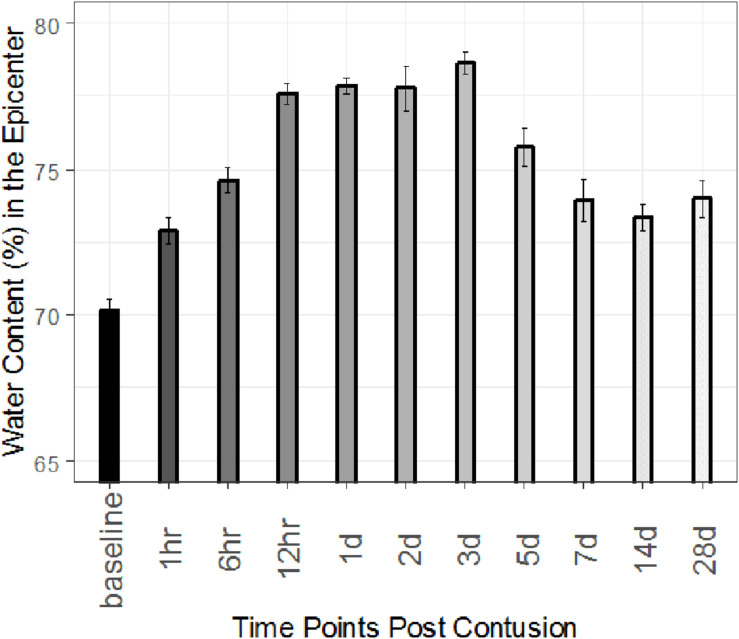
Time course of spinal cord edema following severe SCI at T8 for epicenter. Percent (%) water content calculated in sham rats and injured rats at 1, 6, 12, 24, 48, and 72 h and 5, 7, 14, and 28 days post injury. Error bars indicate standard error (SE). For all times, *n* = 3.

We examined edema progression (percent water content) at 1, 6, 12, 24, 48, 72 h and 5, 7, 14, and 28 days (d) after injury. Figure 2 shows the resulting % water content results at the epicenter. It is seen that water content in the epicenter increases immediately in an hour after contusion. It approaches its peak on day 3 or 72 h post contusion. By the end of the study on day 28, water content is still very higher compared to that at baseline. The numerical values are shown in Supplementary Table S1. These differences are tested using a linear mixed model (Cnaan et al., 1997; Festing and Altman, 2002). It is shown that water content is significantly higher compared to baseline values at 1 h, 3 and 28 days after contusion (2.73, 8.50, and 3.80%, respectively) (Table 1).

**TABLE 1:** Water content comparisons at the four critical stages.

Critical time point comparison	Water content or its difference (%)	95% CI	*P* value
Baseline	70.17	69.01–71.32	<0.0001
Post 1 h vs Baseline	2.73	1.34–4.13	0.003
Post 3 days vs Baseline	8.50	7.11–9.89	<0.0001
Post 28 days vs Baseline	3.80	2.41–5.19	0.0005

In addition, rostral and caudal areas adjacent to the lesion epicenter showed significant increases in water content 24 h after injury, peaking at 72 h before returning to baseline at 7 d. However, water content was only different from its baseline on day 3 after contusion in the rostral and caudal segments. (Rostral and caudal time course Supplementary Figure S1 and Supplementary Tables S2–S5.) Raw data is available in Supplementary Table S6.

### Development of a Spinal Cord OTD

**FIGURE 3:**
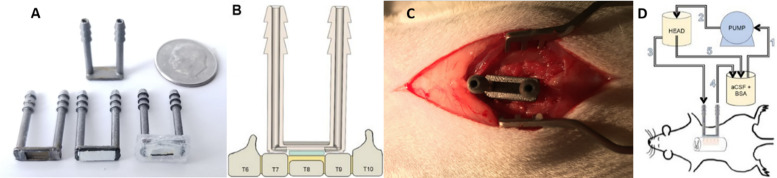
Deployment of the osmotic transport device (OTD) to reduce edema at the site of spinal cord injury. **(A)** Basic OTD design that consists of a fluid housing chamber with a supported semipermeable membrane bottom. The device consists of 19 mm inlet and outlet ports connected by a shallow chamber at the base of the device. The length of the ports allow for ease of access to the device following wound closure. The bottom of the chamber houses the semipermeable membrane (10 kDa cutoff) adhered to its underside that provides a continuous flow channel between ports for fluid and solutes not permeating the membrane. The device has an additional silicone housing to protect the animal and provide additional sealing. Images counterclockwise from the top: top view of OTD without silicone housing; bottom view of OTD without membrane attached or silicone housing; bottom view of OTD with attached membrane; bottom view of OTD with membrane attached and silicone housing. **(B)** Following T8 laminectomy and severe contusion spinal cord injury, the dorsal processes of T7 and T9 are removed and flattened. Hydrogel (green) is then placed on the surface of the exposed injury site (yellow) and the OTD is deployed and sealed with additional silicone. The hydrogel provides continuous fluid continuity between the OTD semipermeable membrane and the tissue. **(C)** Photograph of the OTD deployed in the animal without additional silicone application. **(D)** Aqueous proteinaceous solution (aCSF + BSA, 350 g/L) is delivered from a reservoir (1) through a pump, which is then transferred to a suspended vessel to maintain head pressure (2) via an overflow process (5). The solution is then delivered to the inlet port of the OTD (3) where it passes tangentially across the semipermeable membrane. The BSA is impervious to the membrane and results in an induced osmotic pressure that drives fluid from the tissue into the OTD. The effluent of the OTD is then returned to the beaker (4) where it can once again complete the cycle for continuous treatment.

The device design consists of a flat semi-permeable membrane separations structure that is mounted in a two-compartment housing with two ports that allow tangential flow of an osmotically active fluid across the membrane on one side (Figure 3A). The osmolyte is impervious to the membrane but water and ions can freely cross the barrier. The opposite side of the membrane is loaded with a hydrogel and is placed direct in contact with the tissue at the point of injury (Figure 3B) in the animal (Figure 3C). Details of the device development are in the Supplementary Material section “Membrane Device Design.” Artificial cerebral spinal fluid (aCSF) containing 350 g/L bovine serum albumin (BSA) as the osmolyte is circulated through the device (Figure 3D) for 3 h beginning 1 h after injury. The device is estimated to have an extraction rate on the order of 30 μL/h (see Supplementary Table S7). Following treatment, the animal is sacrificed, and tissue is dissected for analysis of spinal cord % water content.

### Edema Reduction in 3 h SCI Contusion Study

**FIGURE 4:**
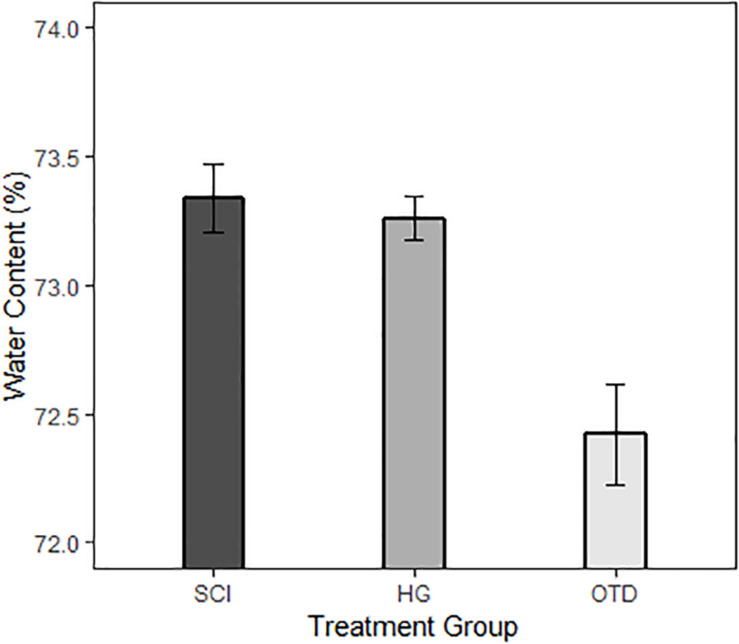
Effects of OTD treatment on % water content after severe SCI at T8. Percent (%) water content calculated SCI only, SCI + hydrogel (HG), and SCI + OTD rats following 3 h of treatment. The figure shows a statistical reduction in % water content in tissue following OTD treatment. Values are shown for 5 mm segments isolated from the lesion epicenter (*n* = 5 for all groups).

Figure 4 shows tissue water content (%) for the treatment groups, injured animals receiving no treatment (SCI), injured animals treated with an inoperable OTD with hydrogel (SCI + HG) and injured animals with the operating OTD (SCI + OTD). Mean and standard error are shown in Table 2. Injury (SCI, *n* = 5) caused an increase in water content to 73.3 ± 0.30%. The (SCI + HG) case had the entire OTD with hydrogel implanted but did not have flow within the device during the observation period. The results for the (SCI + HG, *n* = 5) case did not significantly differ from the injured, untreated case at 73.3 ± 0.19%. This confirms that the non-operational device had no significant impact, indicating that water content reduction was not due to the hydrogel alone. However, the treatment case with a functional OTD (SCI + OTD, *n* = 5) had water content value of 72.4 ± 0.43%. The study results correspond to a reduced tissue water content in OTD treated animals (SCI + OTD) at the lesion epicenter compared to injured, untreated animals (SCI). Water content in the OTD treatment group is remarkably lower than that of SCI group (mean: 73.34% and 95% CI: 73.03–73.65). The treatment effect is −0.92% (95% CI: −1.37 – −0.47%, *p* < 0.0001). However, the treatment effect of HG is not significant (Table 3). For OTD group Cohen’s effect size is 0.49, generally seen as a medium level. Although the OTD did not return the tissue to the uninjured water content, it resulted in approximately a 29% reduction in edema compared to the injured group. The significance of this is illustrated in the section “Discussion.”

**TABLE 2:** Summary on water content in the three treatment groups.

Treatment group	Sample size	Mean (%)	SD	SE
HG	5	73.26	0.19	0.09
OTD	5	72.42	0.43	0.19
SCI	5	73.34	0.30	0.13

**TABLE 3:** Treatment effect estimation using a linear regression model.

Treat group comparison	Group difference in water content (%)	95% CI	*P* value
SCI	73.34	73.03–73.65	<0.0001
OTD vs SCI	–0.92	−1.37 – −0.47	<0.0001
HG vs SCI	–0.08	−0.53–0.37	0.57

## Discussion

### Edema Progression

In this study, we performed a detailed analysis of the time course of spinal cord water content after severe thoracic contusion SCI in the rat model for the first time. At the lesion epicenter, spinal cord water content was significantly elevated as soon as 1 h after injury, peaked at 72 h at a value of (78.7 ± 0.67)%, and remained elevated at 28 d after injury. At segments 5 mm rostral or caudal to the lesion epicenter, spinal cord water content was elevated 1 d after injury, peaked at 72 h, and returned to baseline by 7 d after injury (see Supplementary Figure S1). The total increase in water content during edema progression at the epicenter was 8.5% (up from 70.2% for sham). The sham water content values are consistent with the literature (Sharma and Olsson, 1990). These data suggest that there is a period of approximately 3 days of peak edema spreading from the injury epicenter radially along the parenchyma of the spinal cord, and thus inform the possible “treatment window” needed for therapeutic spinal cord edema reduction.

### Estimated Water Extraction Rate by the OTD

The estimated extraction rate on the order of 30 μL/h for the OTD in the *in vivo* studies indicates that the device can remove substantially more water than that associated with edema. The estimated geometry implies that the excess water is approximately 7.2 μL of fluid. This is substantially less than the 90 μL of fluid expected to be removed during the 3 h operation of the OTD. It is likely that, during significant swelling, the OTD can extract fluid directly from edema in the cord (Figure 1). After the swelling radius has reduced to a critical point, extraction of additional fluid is likely from surrounding tissue and the subarachnoid space.

### Relatively Small Increases in % Water Content Can Result in Vascular Constriction in the Spinal Cord

Relatively small changes in % water content have been shown to be significant in cerebral edema (Keep et al., 2012; McBride et al., 2012). This is also likely in SCI where constriction in the narrow subarachnoid space can lead to vascular compression. The water content measurement can be used to estimate the degree of radial swelling of the cord at the epicenter that could result in vascular constriction in the subarachnoid space. Using estimates of the spinal cord dimensions and water content results, we developed a first approximation model of spinal cord swelling with respect to water content (illustrated in Figure 1). The spinal cord is approximated as a uniform cylindrical tube with swelling due to edema represented as a centrally located spherical element. The uninjured volume, *V*_*i*_, is then

(1) $$V_{i}=\pi \,L\,R_{i}^{2}$$

where *L* is the length and *R*_*i*_ is the initial radius of the spinal cord. The additional increase in volume, *V*_*a*_, caused by swelling is

(2) $$V_{a}=\frac{4\,\pi }{3}\,{\left(R_{s}^{2}-R_{i}^{2}\right)}^{1.5}$$

where *R*_*s*_ is the swollen radius of the spinal cord. Given the initial and final percent water content and assuming constant density of the fluid associated with the spinal cord, the radius due to swelling can be determined by iteration using the relationship,

(3) $$\frac{\%water_{f\,i\,n\,a\,l}}{\%water_{i\,n\,i\,t\,i\,a\,l}}=\frac{4\,{\left(R_{s}^{2}-R_{i}^{2}\right)}^{3/2}}{3\,R_{i}^{2}\,L}+1.$$

**FIGURE 5:**
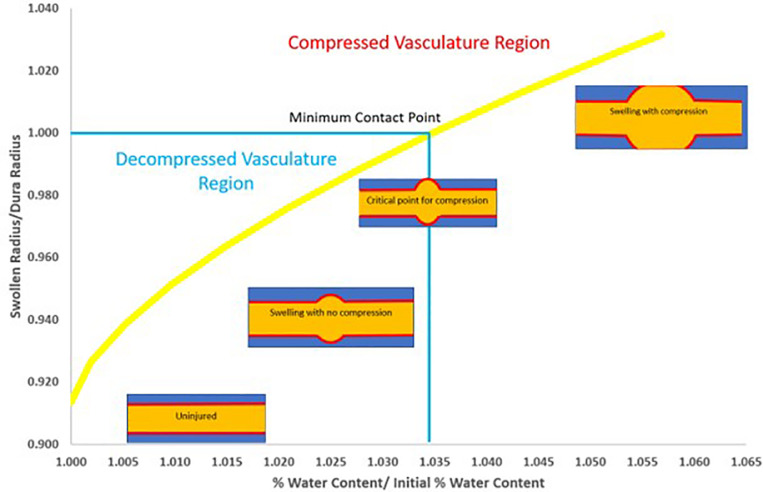
Calculated illustration of relationship between changes in % water content and potential subarachnoid and vascular compression due to radius swelling. Graphic is based on a typical uninjured cord radius of 1.48 mm (Soubeyrand et al., 2014a), an overall radius including the subarachnoid space of 1.62 mm (Soubeyrand et al., 2014a) (radii ratio of 0.913), a 5 mm segment and an uninjured spinal cord water content of 69.4% (normalized as 1.0% water content/initial % water content). Based on measured size of the excised tissue in this study and the assumption of spherical swelling in a cylindrical vessel, a % water content ratio increase of only 0.035 (equivalent to a % water content increase of 71.8%) results in a swelling radius at the threshold for constriction of the subarachnoid space and potential collapse of the local vasculature (radii ratio of 1.0). Although this is only an estimate, nevertheless, these results imply that even relatively small reductions in edema may support reduced vascular compression that may help improve recovery.

Figure 5 illustrates the significance of changes in water content to potential vascular constriction for radial swelling at the epicenter for a 5 mm segment of a model rat spinal cord. In this illustration, radial dimensions for the cord (1,480 μm) and subarachnoid space (1,619 μm) are estimated from very high resolution ultrasound images of Wistar rat spinal cord (Soubeyrand et al., 2014a). Using water content results from this study and the Wistar rat spinal cord dimensions, only a 0.035 decrease in water content ratio (or decrease from 71.8 to 69.4% water content) is required for predicted decompression of the subarachnoid space and hypothetically reduce constriction of the local vasculature. While the spinal cord and subarachnoid space are clearly non-uniform, this estimate addresses how minute increases in % water content due to swelling may lead to constriction of the local vasculature in SCI. This is consistent with experimental results observed by others (Saadoun and Papadopoulos, 2020).

### OTD Has Potential Therapeutic Benefits

The comparison between the calculated water content for the threshold for edema water and the value in which the OTD can reduce the water content is remarkably similar, albeit, the direct dimensions of the spinal cord tissue used here have not been determined for the Sprague Dawley rats used in this study. This result implies two important insights: (1) the OTD may reduce swelling to a level of therapeutic significance, and (2) there may be significant therapeutic benefits from reducing the water content by even a relatively small percent. The results from this study shows that the reduction of edema by the OTD (from 73.3 ± 0.30% to 72.4 ± 0.43%) can potentially reducing vascular collapse and opening the subarachnoid space. It is noteworthy, however, that our device functions by removal of water content from the spinal cord through the dura. We anticipate that a severe spinal cord contusion using the IH Impactor (Infinite Horizons impactor, model # IH-0400, Precision Systems and Instrumentation, LLC) may disrupt the collagen and elastin fibers that make up the dura, allowing for water extraction through a disrupted water-tight barrier (Maikos et al., 2008; Soubeyrand et al., 2014b). Assessment of dural integrity following contusion injury will be necessary to determine the mechanism of action of our current approach, as well as the long-term viability of above dural treatments. Further, investigation into ISP, SCPP and intraoperative ultrasound imaging to verify vascularity and metabolic state of the tissue following OTD treatment are necessary to further validate our theoretical model and identify the therapeutic potential of this novel approach.

### Scaling to Human Parameters

This analysis can scale to human parameters. We estimate that the OTD can perform well above the therapeutic limit for its application in patients. Assume that swelling volume scales with cord radii and the available surface area of the cord to deploy the OTD increases by an order of magnitude for human. Then assuming the overall hydraulic resistance through the human dura is no more than an order of magnitude of the rat, the effective removal rate for a human would be approximately 90 μL in 3 h, based on our computational studies. In addition, the above studies were performed with the relatively low osmotic pressures, which can be dynamically controlled in the OTD if necessary.

## Methods

### OTD Development

#### Design Considerations

The device is primarily structured with a tangential flow module supporting a semipermeable membrane. The membrane is in contact with a hydrogel that rests on the exposed tissue. aCSF containing a rejected osmolyte is passed across the solution side of the membrane. At the membrane surface, the osmolyte in the OTD initiates controlled fluid removal from the tissue where it is expelled with the effluent.

Excess water removal by the OTD requires fluid permeability across the dura mater as well as other tissue between the OTD and the spinal cord core. As shown in Figure 1, the flux through the OTD must pass in series through the hydratable material and through the semipermeable membranes. Using the Kedem–Katchalsky model for membrane processes, the water flux, *J*_*v*_, through the device, for a uniform transport area is described as (Kedem and Katchalsky, 1958):

(4) $$J_{v}=\frac{1}{\mu }\,\frac{\Delta \,P-\sigma_{B\,S\,A}\,\Delta \,\pi_{B\,S\,A}-\sigma_{o\,t\,h\,e\,r}\,\Delta \,\pi_{o\,t\,h\,e\,r}}{R_{m}+R_{m\,e\,m\,b\,r\,a\,n\,e\,s\,u\,p\,p\,o\,r\,t}+R_{h\,y\,d\,r\,o\,g\,e\,l}+R_{d\,u\,r\,a}+R_{p\,i\,a}}$$

where Δ*P* is the transmembrane pressure driving force, *Δπ* is the osmotic pressure, *σ* is the osmotic reflection coefficient which provides a measure of the membrane permselectivity (approximately unity in our studies), *R*_*m*_ is the membrane resistance, *R*_*membrane  support*_ is the flux resistance due to the membrane support, *R*_*hydrogel*_ is the hydrateable hydrogel resistance, *R*_*dura*_ is the hydraulic resistance due to the dura mater tissue, *R*_*pia*_ is the resistance to the pia mater tissue, and μ is the solution viscosity.

The OTD operates as a standard membrane process except Δ*P* < Δπ is required to obtain a negative *J*_*v*_. This is accomplished using low flowrates so that the flux of solvent is into the OTD and away from the tissue. However, in operation, permeating fluid passing through the membrane dilutes the osmolyte at the membrane surface. Since the governing osmotic pressure is associated with the osmolyte concentration immediately at the membrane surface, a computational fluid dynamics model (COMSOL Multiphysics, COMSOL, Inc., Burlington, MA, United States) was used to estimate the osmotic pressure relative to the internal tangential flow inside the OTD and the resulting permeate flux during operation. Details of the modeling approach are illustrated in the Supplementary Material section “Computational Modeling of Device Efficacy.”

### OTD Operation

The solution chosen was 350 g/L BSA (65,000 MW) solution in 0.15 M salt aCSF at pH 7.4. To prepare the solution, aCSF solvent was used to dissolve a weighed amount of BSA (RPI, A30075-100.0X). The solution pH was adjusted using 1 M HCl and 1 M NaOH while undergoing stirring to prevent local denaturation of BSA. The volume of acid and base used to adjust pH was considered part of the solution and was accounted for when determining concentration. The volume of solution considered the specific volume of protein and salt. The computational estimate of the osmotic pressure across the tissue and the membrane was 11.3 kPa (see Supplementary Material).

A Microdyne Nadir, Spectra/Por^®^3 10 kDa polyethersulfone (PES) membrane with a support backing of hydrophilic polyethersulfone (PESH) was used for the device membrane. The membrane was chosen for its hydrophilic nature and its rejection of the osmotic agent.

The hydrogel used in this work is 0.3% agar (Sigma, 05040-1KG), by weight, dissolved in aCSF solvent. The agar/aCSF solution was placed in a container to achieve the proper gel height. Next the solution was heated for 30 s in a microwave set to high. Agar was chosen due to its biocompatibility (Tonda-Turo et al., 2017). Although the water content is higher than that of the tissue, the watery consistency of the gel insures that the device maintains contact with the tissue.

#### Selected Operating Conditions

The process was operated with a fixed head pressure of 2.9 kPa to ensure a negative flux (Figure 3D). This resulted in an operating flowrate tangential to the membrane of 25 μL/min. We determined the initial osmotic pressure of 350 g/L BSA in aCSF to be 131.4 kPa. The estimated osmotic pressure during operation was reduced to approximately 8 kPa. The conservative estimated overall permeate flux across the membrane was determined to be on the order of 30 μL/h. We recently demonstrated with densimetry methods that the computational analysis was consistent with experimental observation (Hale et al., 2019). These results were determined by CFD model calculations and a 50% reduction in flux due to the expected resistance from the dura and pia tissue (see Supplementary Table S8).

### Ethics

All experiments were performed with approval from the University of California Animal Care and Use Committee and in accordance with the National Institutes of Health Animal Care and Use Guidelines, AUP# 2018-0011.

### Animals

Female Sprague Dawley (SD) rats were purchased from Charles River Laboratories. Animals were maintained under a 12-h light/dark cycle and were provided food and water *ad libitum*. All rats were 8–10 weeks of age at the time of experimentation. A group size of three was used for each time point for the edema progression study and five animals were used for each case in the treatment study. The sample size of the animals is calculated in terms of a resource equation (Kilkenny et al., 2009).

### Spinal Cord Injury

Rats were anesthetized with isoflurane inhalation and given an intraperitoneal injection of ketamine and xylazine (K/X) (80/10 mg/kg). We evolved toward isoflurane induction then used ketamine/xylazine injection anesthesia to avoid hemorrhage. With this regimen, we were able to get (1) a reproducible and titratable level of anesthesia appropriate for these experiments; (2) lack of motion of the spine/spinal cord during device application; and (3) lack of hemorrhage. This method also insured that any effect on hemodynamics would be similar across mice given the same anesthetic regimen.

Rats were aseptically prepared for surgery and artificial tear ointment was applied to the eyes to prevent drying. Toe pinch reflex was used to measure anesthetic depth every 10 min throughout the surgery, and supplemental doses of K/X were administered, as needed. A midline incision 2–3 cm long was made along the dorsal surface of the animal and overlying muscle was separated to allow visualization of the spinal column. A laminectomy was performed at thoracic level 8 (T8). For the injury groups, the Infinite Horizons (IH) impactor (Infinite Horizons impactor, model # IH-0400, Precision Systems and Instrumentation, LLC) was used to produce a severe contusion injury of the spinal cord. The exposed cord was contused with a 250 kilodyne (kD) force using a 2.5 mm probe centered along the dorsal column using standard methods (Scheff et al., 2003; Moreno-Manzano et al., 2009; Beggs et al., 2015). Example impact statistics are shown in the Supplementary Figure S9. Control animals received a laminectomy only. Following impact, the cord was examined for adequate bilateral bruising, overlying vertebral muscles were closed with 5-0 chromic gut sutures and skin was closed with 9 mm wound clips.

### Device Mounting

For animals receiving OTD placement, spinal cord exposure and injuries were produced as previously described. Following laminectomy and/or contusion injury, the dorsal processes of the T7 and T9 lamina were removed and flattened to accommodate the length of the device and allow direct contact between the OTD and the underlying tissue at T8 (Figures 3B,C). Following device placement, the overlying vertebral muscles were closed with 5-0 chromic gut sutures and skin was closed with wound clips.

### Post-operative Care

Post-operative care was performed on animals included in the edema time course. Post-operatively, rats were placed on alpha-dri bedding on a 37°C water jacket to maintain adequate body temperature. Rats were monitored daily for general health, mobility in the cage, adequate feeding, proper hydration, and signs of distress, including weight loss, piloerection, and porphyrin. Animals were given lactated ringers (5 ml/100 g) for hydration and baytril (5 mg/kg) to prevent infection for 7 days following injury. Animals received buprenorphine (0.5 mg/kg) immediately after surgery and 4 h post-surgery. Buprenorphine administration was continued two times per day (every 12 h) for another 3 days post-surgery. Finally, animals underwent manual bladder expression until bladder function was recovered (typically within 1–2 weeks post injury).

### Water Content

At each experimental endpoint, animals were sacrificed with Fatal Plus (100 mg/kg given I.P.) followed by cardiac puncture, after which 5 mm of spinal cord centered at the injury epicenter, as well as rostral and caudal to the injury (15 mm total), were rapidly dissected and assessed for spinal cord water content. Freshly dissected tissue was placed on a pre-weighted piece of foil and the tissue weight was recorded. Tissue was then dried in an oven at 85°C for 48 h and reweighed. Percent water content was calculated as (wet weight − dry weight)/wet weight × 100. This method allowed for a measure of edema within and immediately surrounding the lesion site.

## Data Availability Statement

All datasets presented in this study are included in the article/Supplementary Material.

## Ethics Statement

The animal study was reviewed and approved by University of California Animal Care and Use Committee, AUP# 2018-0011.

## Author Contributions

CH designed and produced device, prepared computational analysis, supported experimental procedure, helped to write the manuscript, and equal contribution. JY performed *in vivo* experiments, supported experimental procedure, helped to write the manuscript, and equal contribution. RB performed osmotic pressure experiments. RC built OTD devices. CJ helped to write the manuscript. DB conceived this work, supervised *in vivo* work, and helped to prepare the manuscript. VR conceived this work, supervised device development, developed modeling analysis, and prepared the manuscript. All authors contributed to the article and approved the submitted version.

## Conflict of Interest

The authors declare that the research was conducted in the absence of any commercial or financial relationships that could be construed as a potential conflict of interest.
