# Supplementary material for: Implantable Osmotic Transport Device Can Reduce Edema After Severe Contusion Spinal Cord Injury
Source: Front Bioeng Biotechnol. 2020 Jul 10;8:806. doi: 10.3389/fbioe.2020.00806 (PMC7366393; doi:10.3389/fbioe.2020.00806)
Supplement: Supplementary file 1 [file Data_Sheet_1.docx]

# Contents

[Contents 1](#_Toc38187385)

[S1. Time Course for Edema at Epicenter 2](#_Toc38187386)

[S2. Time Course for Edema at Rostral and Caudal Regions 3](#_Toc38187387)

[S3. Membrane Device Design 7](#_Toc38187388)

[S4. Computational Modeling of Device Efficacy 8](#_Toc38187389)

[S5. Osmotic Pressure Data for BSA in aCSF 16](#_Toc38187390)

[S6. Example Impactor Output 18](#_Toc38187391)

[Reference 19](#_Toc38187392)

# S1. Time Course for Edema at Epicenter

**Table S1.** Water content summary in the epicenter

| Time points | Sample  size | Mean | SD | SE |
| --- | --- | --- | --- | --- |
| Baseline | 3 | 70.17 | 0.61 | 0.35 |
| 1 hr | 3 | 72.90 | 0.79 | 0.46 |
| 6 hr | 3 | 74.60 | 0.72 | 0.42 |
| 12 hr | 3 | 77.57 | 0.64 | 0.37 |
| 1 d | 3 | 77.87 | 0.42 | 0.24 |
| 2d | 3 | 77.77 | 1.33 | 0.77 |
| 3d | 3 | 78.67 | 0.67 | 0.38 |
| 5d | 3 | 75.77 | 1.18 | 0.68 |
| 7d | 3 | 73.93 | 1.22 | 0.71 |
| 14d | 3 | 73.37 | 0.81 | 0.47 |
| 28d | 3 | 73.97 | 1.10 | 0.64 |

# S2. Time Course for Edema at Rostral and Caudal Regions

**Figure S1. Time course of edema for rostral (above), and caudal (below).**

**Table S2.** Water content summary in the rostral segment

| Time points | Sample size | Mean | sd | se |
| --- | --- | --- | --- | --- |
| Baseline | 3 | 69.97 | 0.97 | 0.56 |
| 1 hr | 3 | 68.33 | 0.86 | 0.50 |
| 6 hr | 3 | 70.80 | 1.11 | 0.64 |
| 12 hr | 3 | 70.83 | 0.57 | 0.33 |
| 1 d | 3 | 73.23 | 0.64 | 0.37 |
| 2d | 3 | 72.97 | 2.00 | 1.16 |
| 3d | 3 | 73.43 | 2.65 | 1.53 |
| 5d | 3 | 68.27 | 0.72 | 0.42 |
| 7d | 3 | 67.63 | 0.57 | 0.33 |
| 14d | 3 | 68.30 | 0.10 | 0.06 |
| 28d | 3 | 69.00 | 0.72 | 0.42 |

**Table S3**. Water content summary in the caudal segment

| Time points | Sample size | Mean | sd | se |
| --- | --- | --- | --- | --- |
| Baseline | 3 | 69.90 | 1.51 | 0.87 |
| 1 hr | 3 | 70.43 | 0.85 | 0.49 |
| 6 hr | 3 | 72.17 | 0.68 | 0.39 |
| 12 hr | 3 | 72.93 | 0.78 | 0.45 |
| 1 d | 3 | 74.37 | 1.12 | 0.64 |
| 2d | 3 | 74.13 | 1.59 | 0.92 |
| 3d | 3 | 74.83 | 1.44 | 0.83 |
| 5d | 3 | 70.07 | 2.34 | 1.35 |
| 7d | 3 | 70.87 | 2.49 | 1.44 |
| 14d | 3 | 69.43 | 0.45 | 0.26 |
| 28d | 3 | 71.37 | 1.27 | 0.73 |

**Table S4.** Water content comparisons in the rostral segment at the 4 critical stages

| Critical time point comparison | Water content or its difference (%) | 95% CI | P value |
| --- | --- | --- | --- |
| Baseline | 69.97 | 69.01 – 71.32 | <0.0001 |
| Post 1 hr. vs. Baseline | -1.63 | -4.66 – 1.40 | 0.24 |
| Post 3 days vs. Baseline | 3.47 | 0.44 – 6.50 | 0.03 |
| Post 28 days vs. Baseline | -0.97 | -4.00 – 2.06 | 0.46 |

**Table S5.** Water content comparisons in the caudal segment at the 4 critical stages

| Critical time point comparison | Water content or its difference (%) | 95% CI | P value |
| --- | --- | --- | --- |
| Baseline | 69.90 | 68.08 – 71.72 | <0.0001 |
| Post 1 hr. vs. Baseline | 0.53 | -2.05 – 3.11 | 0.63 |
| Post 3 days vs. Baseline | 4.93 | 2.35 – 7.51 | 0.003 |
| Post 28 days vs. Baseline | 1.47 | -1.11 – 4.05 | 0.21 |

**Table S6.** Data used in edema progression analysis

# S3. Membrane Device Design


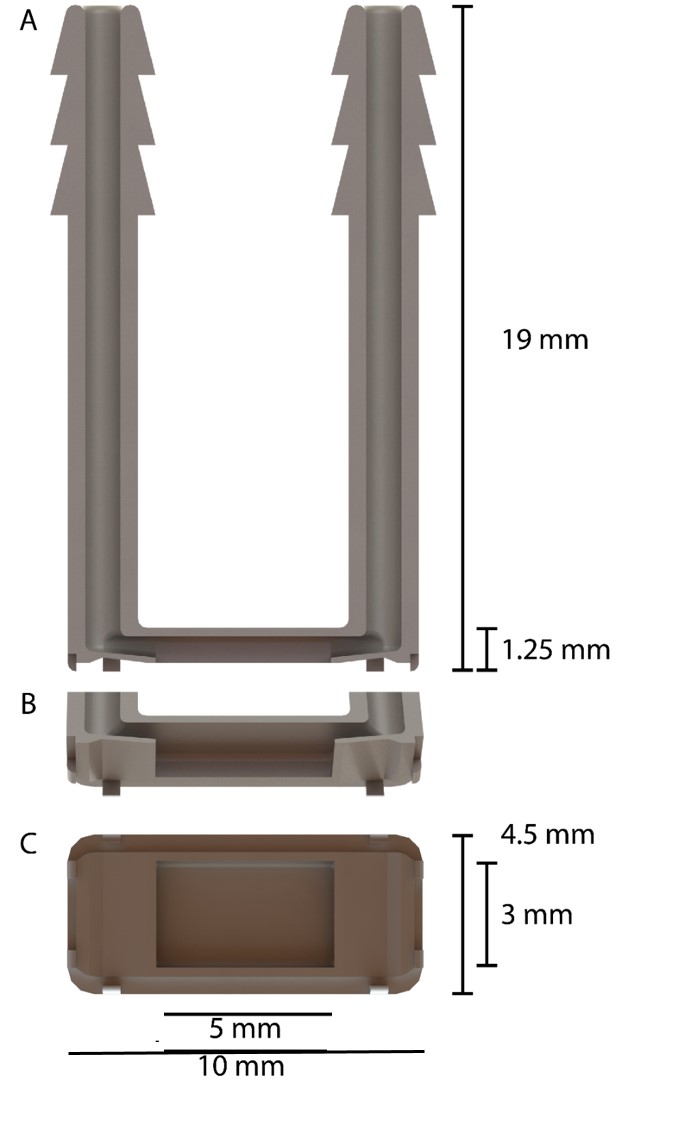


**Figure S2: Images of the membrane device from the front (A), isometric (B), and from the bottom (C).** (Designed using SolidWorks.)

# S4. Computational Modeling of Device Efficacy

Below are the input parameters I used in the COMSOL model.

Inflow: 25 μL min^-1^

Protein concentration: 350 g L^-1^

Membrane hydraulic permeability: 1 x10^-7 [^m/(s-kPa)]

BSA Diffusion Coefficient: 5.9 x 10^-11^[m^2^/s] (Arunyawongsakorn et al., 1985)


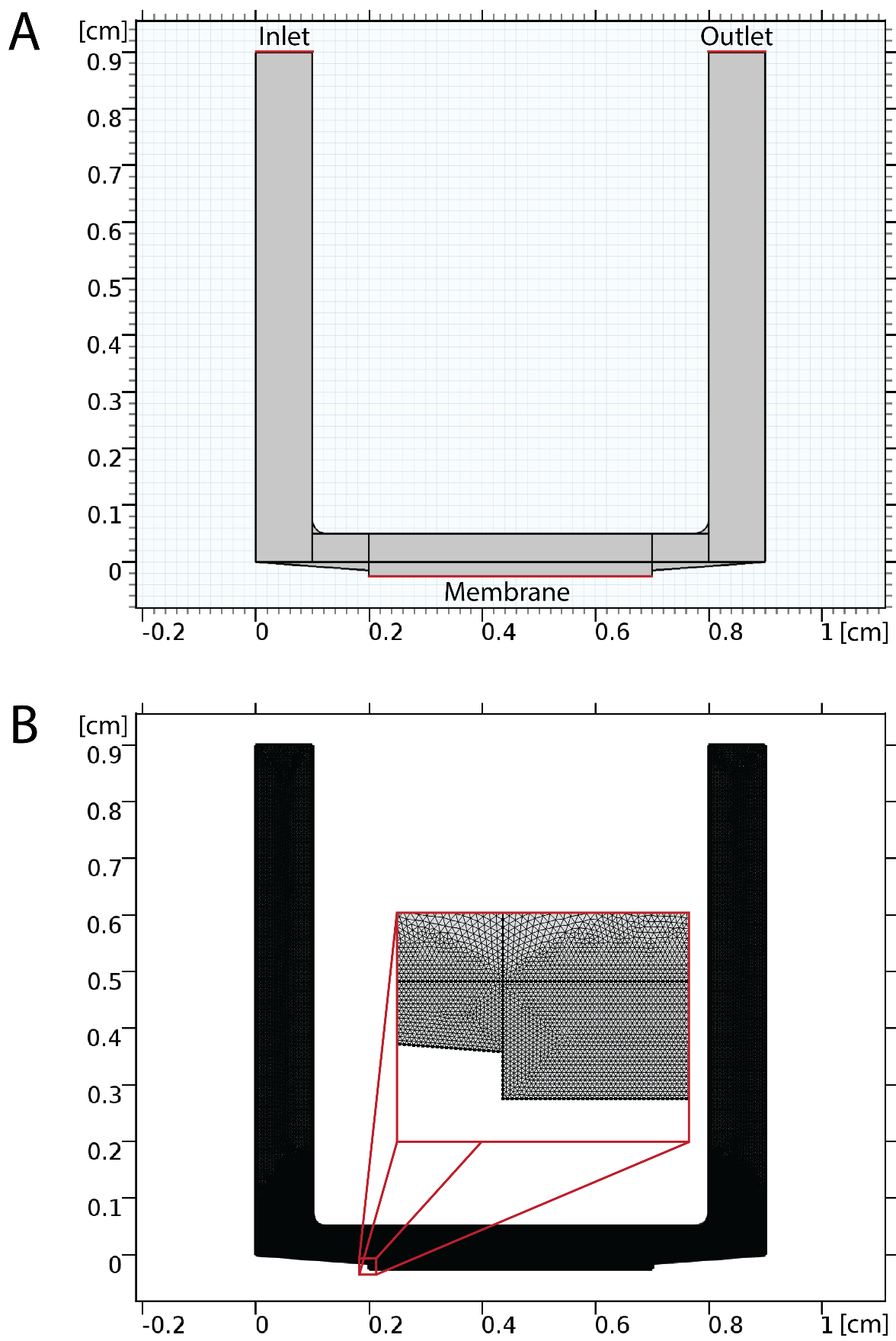


**Figure S3: The membrane device geometry. (A)** modelled in COMSOL. The inlet is in the upper left of the geometry and the outlet is in the upper right with the membrane positioned at the middle bottom of the geometry. Meshing, with a maximum mesh size of 1 µm, is shown in **(B)**.


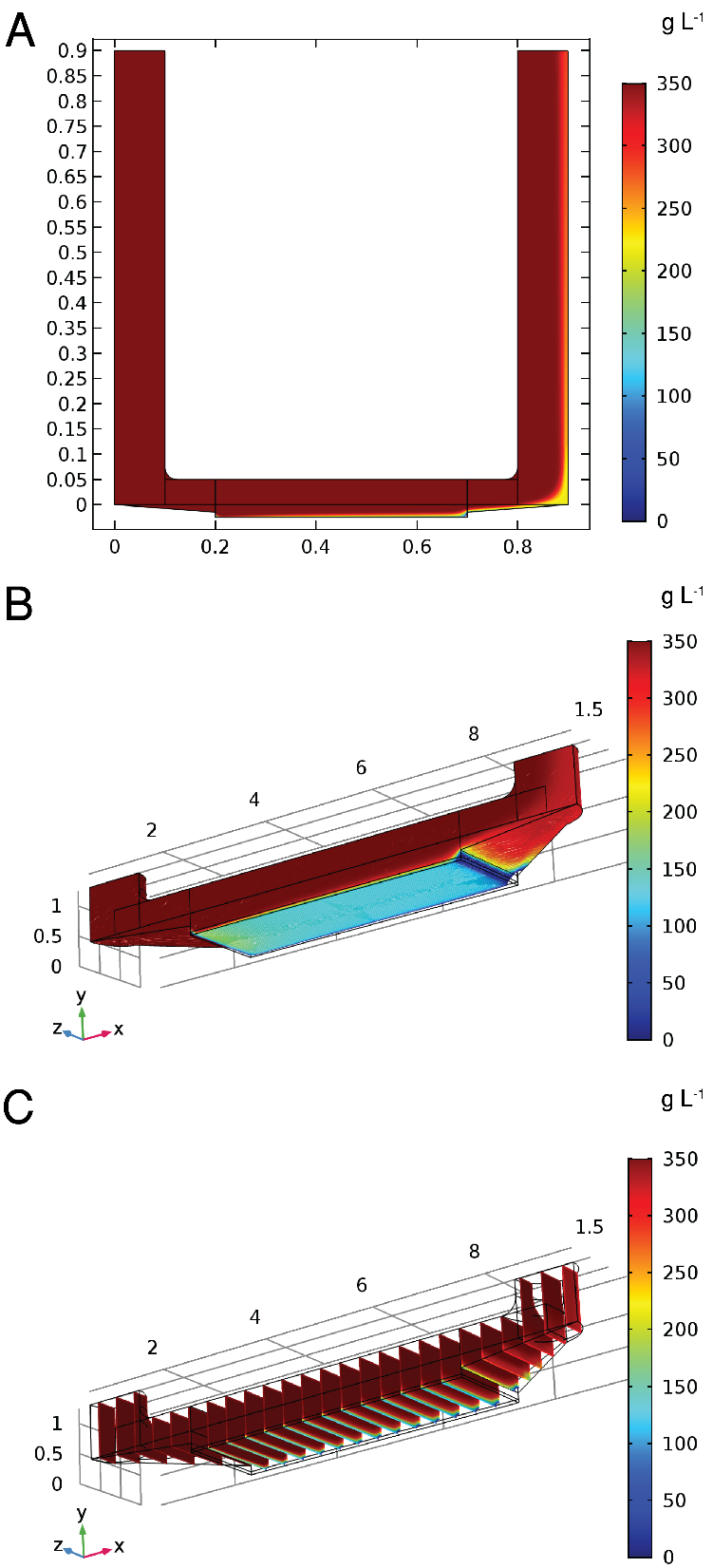


**Figure S4: Concentration profile of the membrane device given an inflow of 25 μL min^-1^ and a protein concentration of 350 g L^-1^.** Dilution is noticeable in regions near the membrane of the profiles.

**Table S7**: Simulated Extraction Rate Dependence on Inlet Flowrate for Membrane Device with Bovine Serum Albumin in Artificial Cerebral Spinal Fluid at pH 7.4, 25°C

| Inlet Flowrate  (µL min^-1^) | Extraction Rate (µL h^-1^) |
| --- | --- |
| 3 | 38.1 |
| 5 | 44.1 |
| 8 | 47.9 |
| 10 | 50.9 |
| 10 | 50.9 |
| 15 | 55.2 |
| 20 | 58.4 |
| 25 | 61.0 |
| 50 | 69.4 |
| 75 | 74.3 |
| 100 | 77.8 |
| 125 | 80.4 |
| 150 | 82.4 |
| 175 | 84.0 |
| 200 | 85.3 |
| 225 | 86.3 |
| 250 | 87.2 |
| 275 | 87.9 |
| 300 | 88.6 |
| 325 | 89.1 |
| 350 | 89.5 |
| 375 | 89.8 |
| 400 | 90.1 |
| 425 | 90.4 |
| 450 | 90.5 |
| 475 | 90.7 |
| 500 | 90.8 |


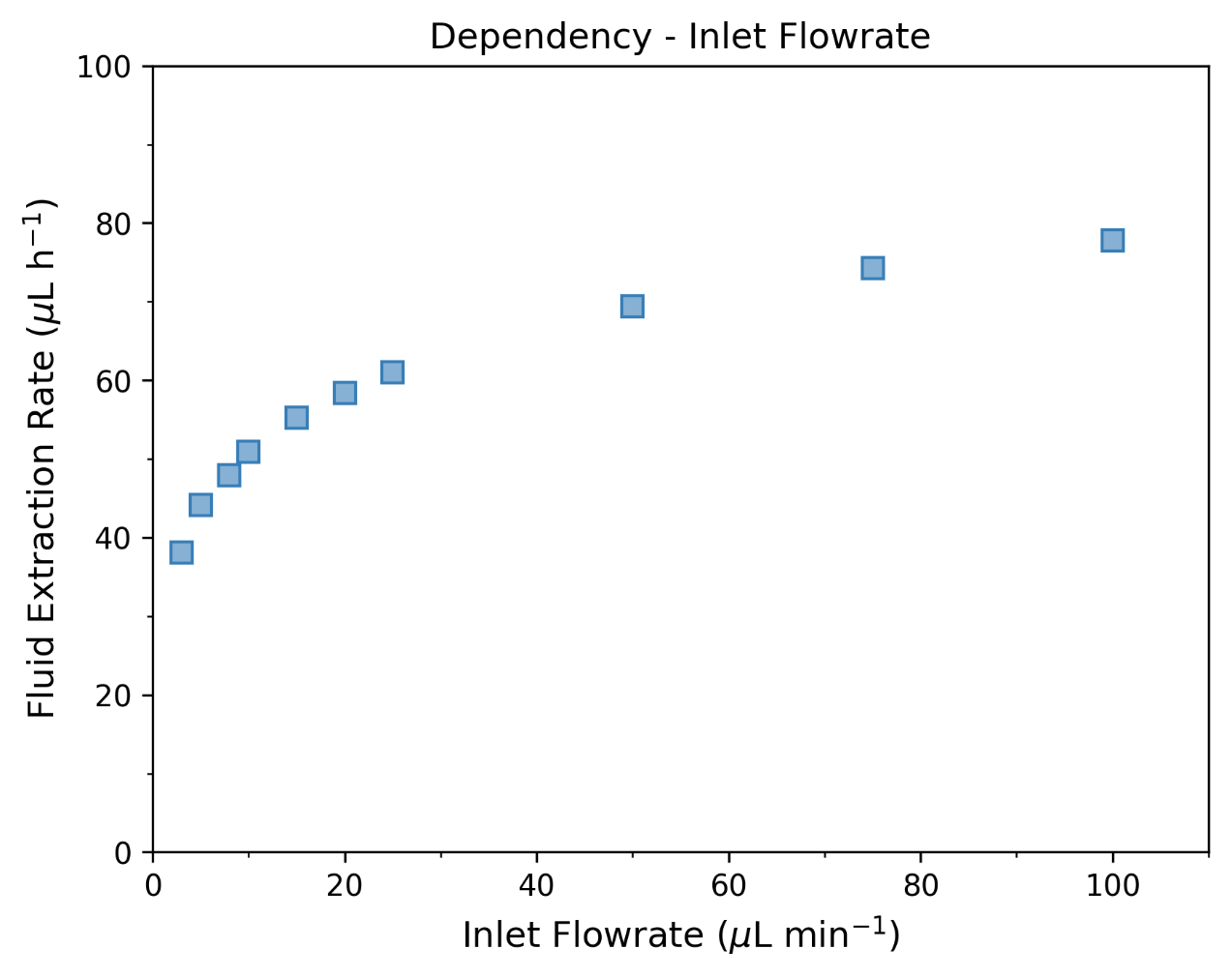


**Figure S5: Computational analysis of fluid extraction rate dependency on inlet flowrate given protein concentration of 350 g L^-1^.** Fluid extraction rate was calculated via the determined velocity through the membrane and the membrane surface area.


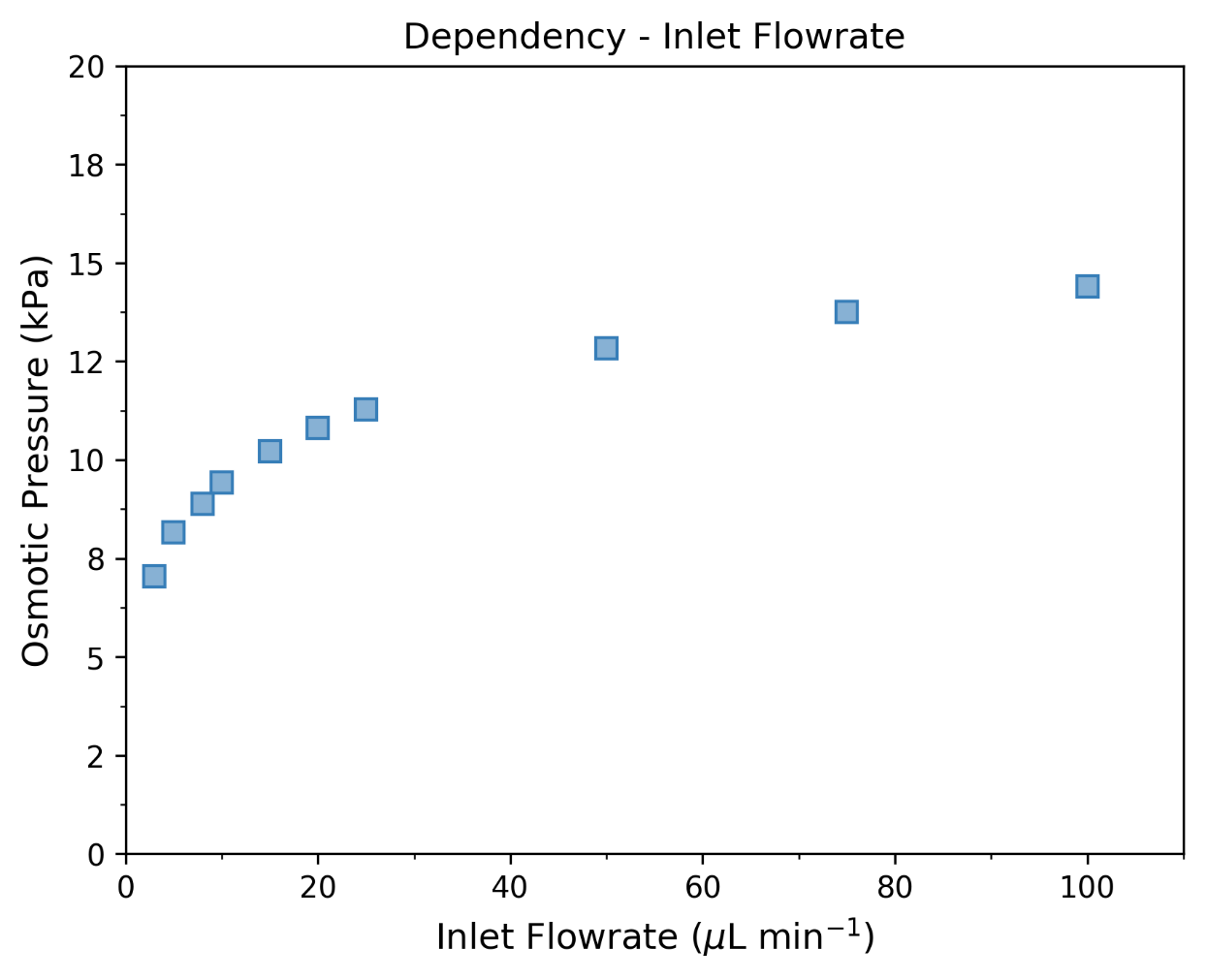


**Figure S6: Computational analysis of the average osmotic pressure at the membrane surface dependent on inlet flowrate given protein concentration of 350 g L^-1^.**

**Table S8:** Simulated Extraction Rate Dependence on Maximum Mesh Size for Membrane Device with an Inlet Flowrate of 25 µL min^-1^ of Bovine Serum Albumin in Artificial Cerebral Spinal Fluid at pH 7.4, 25°C

| Maximum Mesh Size  (µm) | Extraction Rate  (µL h^-1^) |
| --- | --- |
| 10 | 69.82 |
| 9 | 70.81 |
| 8 | 70.73 |
| 7 | 69.81 |
| 6 | 64.11 |
| 5 | 63.77 |
| 4 | 64.17 |
| 3 | 61.84 |
| 2 | 61.28 |
| 1 | 60.99 |
| 0.9 | 60.99 |
| 0.8 | 60.99 |
| 0.7 | 60.99 |
| 0.6 | 61.00 |
| 0.5 | 61.01 |
| 0.4 | 61.04 |
| 0.3 | 61.08 |

Mesh size of 1 µm was used in this study. Extraction rates for actual transport through tissue are reduced by a factor of two to project additional permeate resistances. Thus, the conservative estimate of 30 µL/h is used in this study.


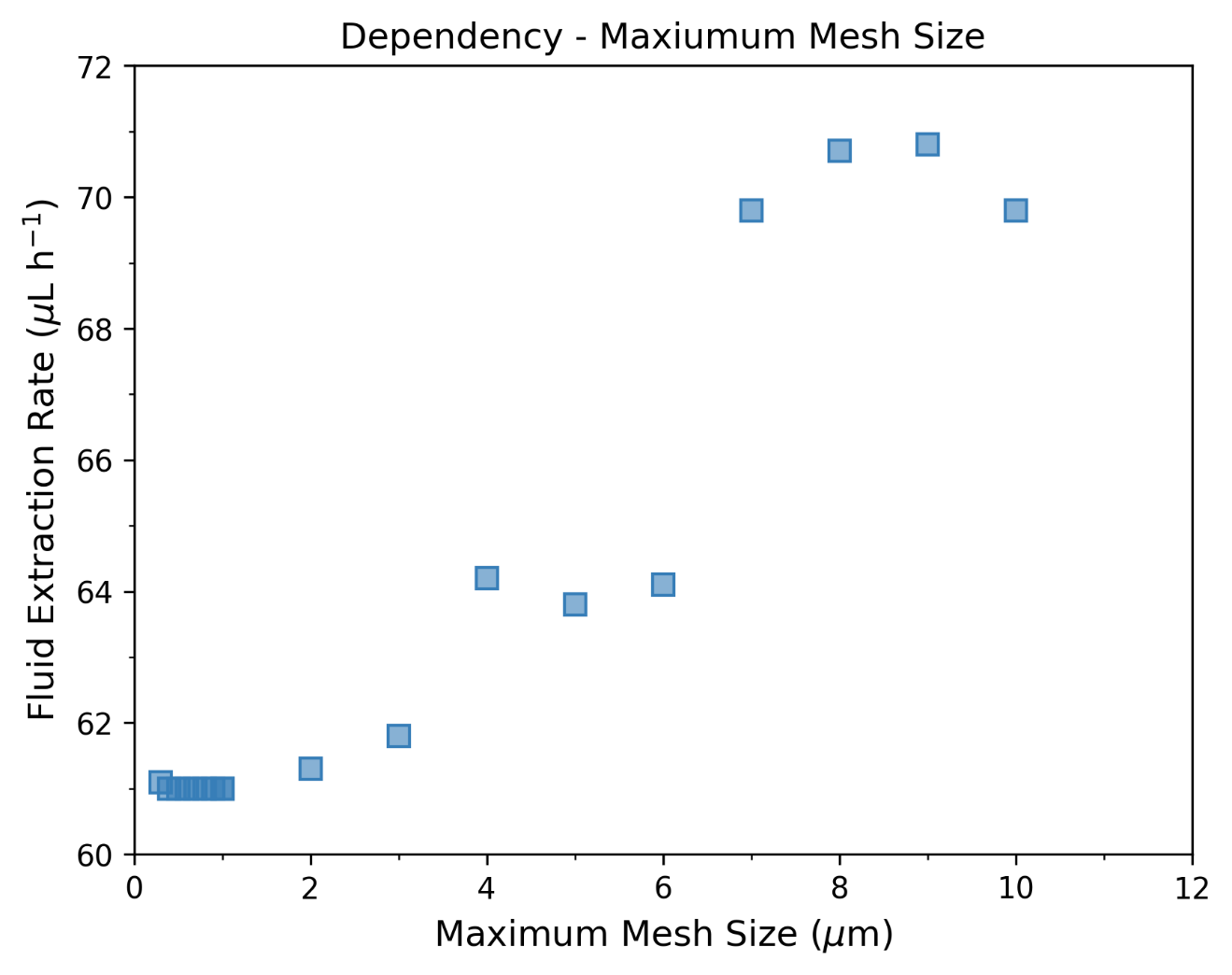


**Figure S7: Evaluation of maximum mesh size to extraction rate at a protein concentration of 350 gL^-1^ and an inlet flowrate 25 μL min^-1^.** Mesh independence is reached at 1 μm. The maximum mesh size was 1 μm and contained 285,973 degrees of freedom with 5,444 internal degrees of freedom (Figure S3). Independence from mesh size was determined by evaluating the dependency of extraction rate on maximum mesh size (Table S8, Figure S7). Mesh independence, less than 1% deviation of extraction rate, was determined to occur below 1 μm maximum mesh size.

# S5. Osmotic Pressure Data for BSA in aCSF

**Table S9**: Bovine Serum Albumin in Artificial Cerebral Spinal Fluid at pH 7.4, 25°C

| [BSA]  (gL^-1^ Soln) | Osmotic Pressure (kPa) |
| --- | --- |
| 290 | 99.9 |
| 298 | 77.5 |
| 306 | 87.8 |
| 341 | 134.1 |
| 359 | 135.4 |
| 361 | 130.2 |
| 362 | 143.1 |
| 378 | 176.0 |
| 396 | 196.9 |
| 399 | 246.2 |
| 408 | 141.4 |
| 414 | 243.4 |
| 416 | 264.0 |
| 436 | 390.1 |


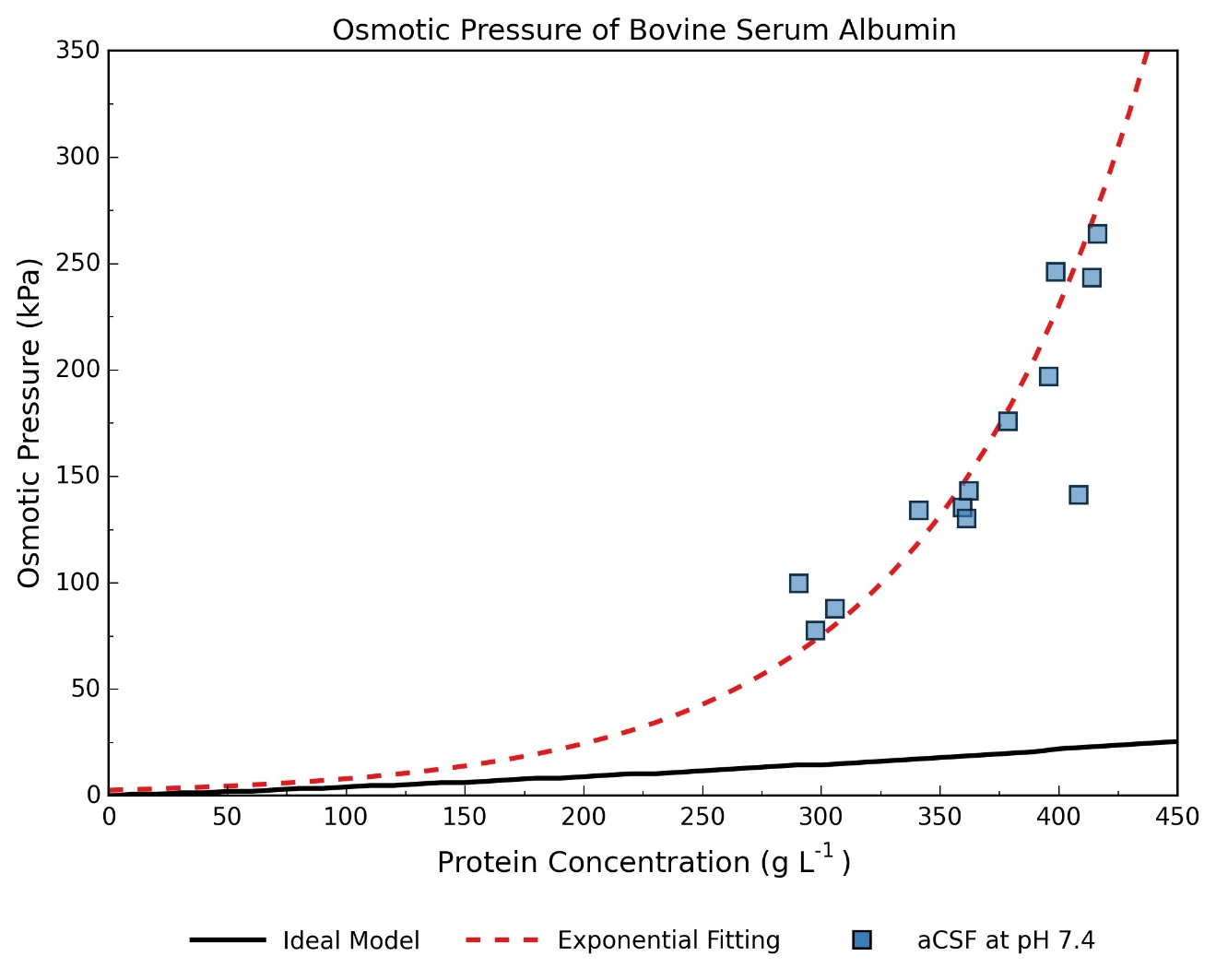


**Figure S8: Osmotic pressure data for bovine serum albumin at pH 7.4 in artificial cerebral spinal fluid.** An ideal model is shown along with an exponential model fitted of the data.

# S6. Example Impactor Output

**Figure S9. Example impactor analysis (time in ms)**


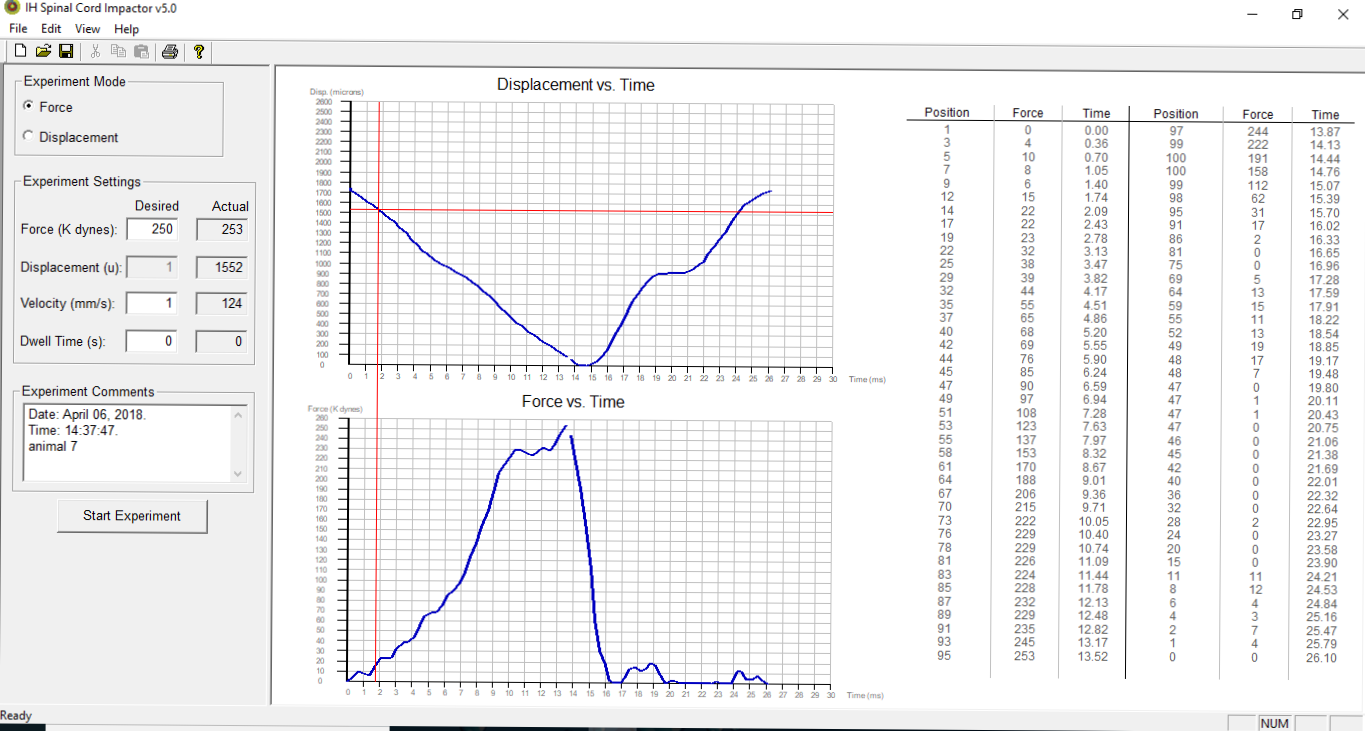


# Reference

Arunyawongsakorn, U., Johnson, C.S., and Gabriel, D.A. (1985). Tracer Diffusion-Coefficients of Proteins by Means of Holographic Relaxation Spectroscopy - Application to Bovine Serum-Albumin. *Analytical Biochemistry* 146(1)**,** 265-270. doi: Doi 10.1016/0003-2697(85)90425-7.
